# Woody encroachment of grasslands: Near‐surface thermal implications assessed through the lens of an astronomical event

**DOI:** 10.1002/ece3.8043

**Published:** 2021-08-27

**Authors:** Evan P. Tanner, Samuel D. Fuhlendorf, John A. Polo, Jacob M. Peterson

**Affiliations:** ^1^ Caesar Kleberg Wildlife Research Institute Texas A&M University‐Kingsville Kingsville TX USA; ^2^ Department of Natural Resource Ecology and Management Oklahoma State University Stillwater OK USA

**Keywords:** grasslands, solar eclipse, temperature, thermal heterogeneity, woody encroachment

## Abstract

Temperature has long been understood as a fundamental condition that influences ecological patterns and processes. Heterogeneity in landscapes that is structured by ultimate (climate) and proximate (vegetation, topography, disturbance events, and land use) forces serve to shape thermal patterns across multiple spatio‐temporal scales. Thermal landscapes of grasslands are likely shifting as woody encroachment fragments these ecosystems and studies quantifying thermal fragmentation in grassland systems resulting from woody encroachment are lacking. We utilized the August 21st, 2017, solar eclipse to mimic a rapid sunrise/sunset event across a landscape characterized as a grassland to experimentally manipulate levels of solar radiation in the system. We then quantified changes in near‐surface temperatures resulting from changes in solar radiation levels during the eclipse. Temperatures were monitored across three grassland pastures in central Oklahoma that were characterized by different densities (low, medium, and high) of *Juniperus virginiana* to understand the impact of woody encroachment on diurnal temperature patterns and thermal heterogeneity in a grassland's thermal landscape. The largest temperature range across sites that occurred during the eclipse was in the mixed grass vegetation. Similarly, the largest change in thermal heterogeneity occurred in the grassland with the lowest amount of woody encroachment. Thermal heterogeneity was lowest in the highly encroached grassland, which also experienced the lowest overall change in thermal heterogeneity during the eclipse. Time series models suggested that solar radiation was the most influential factor in predicting changes in thermal heterogeneity as opposed to ambient temperature alone. These results suggest that highly encroached grasslands may experience lower diurnal variability of temperatures at the cost of a decrease in the overall thermal heterogeneity of that landscape. It appears that fine‐scale spatio‐temporal thermal variation is largely driven by solar radiation, which can be influenced by vegetation heterogeneity inherent within a landscape.

## Graphical abstract

Terrestrial near‐surface thermal conditions are structured by complex ecological patterns that may be facilitated by human‐policy. Using the 2017 total solar eclipse as a natural large‐scale experiment, we investigated rapid changes in patterns of thermal conditions within multiple pastures experiencing varying levels of woody encroachment. Such woody encroachment has predictable and important impacts on thermal landscapes of grasslands, and will have direct implications on the organisms living within these invaded ecosystems.

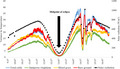

## 1 INTRODUCTION

All living organisms function within the scope of abiotic environmental conditions, which are partially responsible for influencing ecological patterns and processes at multiple spatial and temporal scales (Begon et al., 2006). However, these conditions are highly dynamic in both space and time due to the structural, topographic, and geologic patterns inherent in natural landscapes (Chen et al., 1999; Sears et al., 2011; Turner, 1989). Because of the inherent dynamic state of these conditions, ecological patterns and processes often exist in a state of nonequilibrium (Gilchrist, 1995; Maron et al., 2015; Tanner et al., 2017; Turner, 1989). One such condition, temperature, has long been understood as fundamental in structuring ecological patterns and processes (Angilletta, 2009; Begon et al., 2006). For instance, thermal conditions are important in dictating an organisms’ metabolic rate, which in turn is posited to be a primary constraint that structures biological and ecological patterns globally (Brown et al., 2004). The dynamics of thermal conditions are structured by a complex network of driving forces that are influential from both an ultimate and proximate sense. Ultimately, atmospheric temperatures are influenced by astronomical forces such as incoming solar radiation, while also being governed by many factors such as weather patterns, latitudinal/altitudinal positioning (Ahrens, 2012), and more recently by anthropogenic pressures (IPCC, 2014). Yet in a proximate sense, near‐surface temperatures are governed by interactions between climate and vegetation (Peel et al., 2007), and more locally by patterns of heterogeneity within a landscape that is structured is by soil types, topography, disturbance events, and land use patterns (Sears et al., 2011; Tuff et al., 2016; Turner et al., 2001).

Given the variability of structural heterogeneity inherent in disparate natural and human‐influenced landscapes, coupled with the multi‐scale spatio‐temporal dynamics of weather patterns within these landscapes, the associated thermal landscapes will differ in space and time (Chen et al., 1999; Tanner et al., 2017). A primary determinant of heterogeneity (and subsequently the thermal heterogeneity) within landscapes is vegetation structure and composition, which serve to change near‐surface microclimatic conditions through influencing the amount of direct solar radiation reaching the Earth's surface. However, vegetation can also influence microclimatic conditions through radiative heat transfer (through reflectance, absorption, and transmittance of light waves) and through alteration of wind currents (Geiger, 1965; Stoutjesdijk & Barkman, 1987), all which can be highly dynamic because of structural properties related to individual plants and leaves (Geiger, 1965).

Like near‐surface temperatures, patterns of vegetation structure and composition exist in a dynamic state and are dictated by both natural and anthropogenic factors (i.e., disturbances, soil, and/or land use change [Turner et al., 2001]). Most pronounced in recent decades due to changes in human behavior, anthropogenic factors have acted to alter up to 50%–75% of ice‐free land by changing the ecological processes that historically shaped these systems (Barnosky et al., 2012; Ellis & Ramankutty, 2008). As these anthropogenic pressures alter vegetation patterns, so do they alter the way thermal energy is received at the Earth's surface thus influencing thermal heterogeneity (Tuff et al., 2016).

One such worldwide phenomena related to anthropogenic pressures is that of woody encroachment within grassland systems, which is largely driven by changes in historic fire regimes (Bond, 2008). Such patterns of fragmentation (such as configuration and density of woody cover [Martens et al., 2000]) will significantly impact the resulting thermal landscape (Tuff et al., 2016) and could have important implications for ecological patterns and processes (Smith & Johnson, 2004). Though research has illustrated how such woody encroachment patterns could influence conditions such as solar radiation and soil moisture (Breshears et al., 1997; Martens et al., 2000; Villegas et al., 2010), studies quantifying thermal fragmentation in grassland systems as a result of woody encroachment are lacking (Tuff et al., 2016).

It is likely that the amount of heterogeneity within a landscape is directly correlated with the breadth of an organism's realized thermal niche (Elmore et al., 2017) and that thermally homogeneous landscapes offer fewer opportunities for organisms to maintain optimal body temperatures (Huey et al., 2012). Moreover, temporal variation in thermal conditions can favor the evolution of thermal specialization (Gilchrist, 1995). Fine scale temporal changes in the thermal landscape (i.e., the diurnal temperature range) have been shown to be an important measure of thermal quality for organisms under current and future climatic conditions (Briga & Verhulst, 2015; Oberhauser & Peterson, 2003) and can be directly linked to vegetation structure (Milling et al., 2018). However, linked with such temporal diurnal patterns is a simultaneous change in spatial thermal heterogeneity (the variability in the diurnal temperature range), which can help illustrate the potential a landscape has for allowing certain organisms to maintain optimal body temperatures.

A total solar eclipse offers a unique opportunity to quantify rapid changes in thermal conditions within large‐scale natural landscapes through the dampening of solar radiation that reaches the Earth's surface (Harrison & Hanna, 2016). This phenomenon effectively mimics a rapid sunrise/sunset event (Turner et al., 2018) and facilitates the potential for research on simulated diurnal warming/cooling trends across space and time while minimizing potential effects associated with changes in solar zenith angles that would influence the distribution of sun specks at the surface level. This allows for a rapid assessment of the impact of woody encroachment on diurnal temperature patterns and thermal heterogeneity in grassland thermal landscapes in a standardized approach. It may also help to isolate and quantify the importance of key factors driving thermal variation in landscapes (such diffuse solar radiation) by directly altering levels at a landscape scale. Given the global warming trends projected for future decades (IPCC, 2014) coupled with evidence of unique spatio‐temporal thermal trends such as diurnal asymmetry (Davy et al., 2017), capitalizing on rare astronomical events can help to guide a better understanding of the dynamic nature of thermal landscapes.

During August 21st, 2017, a total solar eclipse known as “The Great American Eclipse” crossed the contiguous United States of America, which began at 9:05 Pacific Standard Time (PST) in Oregon and ended at 14:44 Eastern Standard Time (EST) in South Carolina. This eclipse offered a unique opportunity to study the isolated effects of dampened solar radiation on thermal landscapes, as this was the first total solar eclipse to be visible from the contiguous United States since February 26th, 1979.

The goal of our study was to quantify changes in the thermal landscape in both space and time during this eclipse as they related to a gradient of solar radiation levels (i.e., capturing simulated rapid diurnal temperature and thermal heterogeneity ranges). Furthermore, we sought to relate these changes to patterns of vegetation structure and composition within a grassland matrix with varying levels of woody encroachment. In this study, we had three objectives. First, we sought to (1) quantify changes in temperatures and spatial variance of thermal conditions (Var[*T*]) related to temporal changes in solar radiation during the eclipse. Secondly, we (2) sought to quantify thermal heterogeneity and the range of thermal heterogeneity through varying levels of solar radiation. Finally, we sought to (3) determine the effects of woody encroachment on spatial variance of thermal landscapes within a grassland matrix. Because plant density and area can impact thermal conditions (Geiger, 1965), we predicted that areas of high woody encroachment would have lower spatial variance in temperatures compared to a less fragmented grassland matrix. Furthermore, we predicted that the near complete removal of solar radiation would homogenize spatial variance across vegetation types within the landscape, illustrating the importance that solar radiation plays in structuring thermal spatial variance. To accomplish our goals, we report on the first use of a distributed temperature sensing (DTS) system in a natural landscape during a solar eclipse, which allowed for us to measure thermal conditions as a continuous profile (rather than at discrete point) through the use of fiber optic cables and laser pulse generators.

## 2 MATERIALS AND METHODS

### 2.1 Study area

**FIGURE A1:**
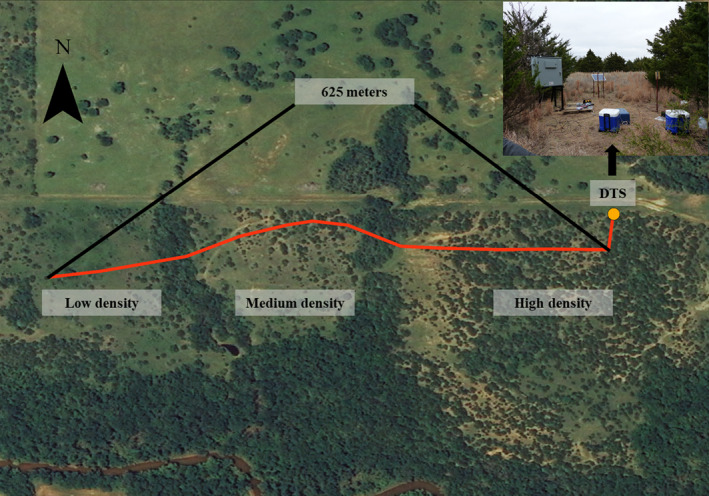
The distributed temperature sensing system (orange circle) and fiber optic cable (red line) were established in a mixed grass prairie with high, medium, and low densities of *Juniperus virginiana* encroachment approximately 15 km west of Stillwater, Oklahoma, USA. A photograph inset illustrates the ambient and cold calibration baths in coolers as well as the weatherproof enclosure that housed the distributed temperature sensing system's controller (gray case). Aerial imagery was obtained from the United States Department of Agriculture's National Agriculture Imagery Program during the 2015 growing season (https://nrcs.app.box.com/v/naip/folder/18020089370)

As part of a long‐term investigation of thermal patterns in southern mixed grass prairies, we located our study approximately 15 km west of Stillwater, Oklahoma, USA. This area represented mixed grass pastures that have experienced encroachment of *Juniperus virginiana* typical of areas that have had fire removed from their system. Our study area had three pastures of varying *J. virginiana* densities, which was quantified as the percentage of our linear cable transect (see next section) that intercepted *J. virginiana* canopies: high (~60% *J. virginiana* cover/cable meter), medium (~46% *J. virginiana* cover/cable meter), and low (~16% *J. virginiana* cover/cable; Figure A1). The climate of our study site was described as continental temperate with long‐term (1981–2010) average August temperatures of 27.2℃ and long‐term average annual precipitation of 93 cm (Brock et al., 1995; McPherson et al., 2007).

### 2.2 Distributed temperature sensing

**FIGURE A2:**
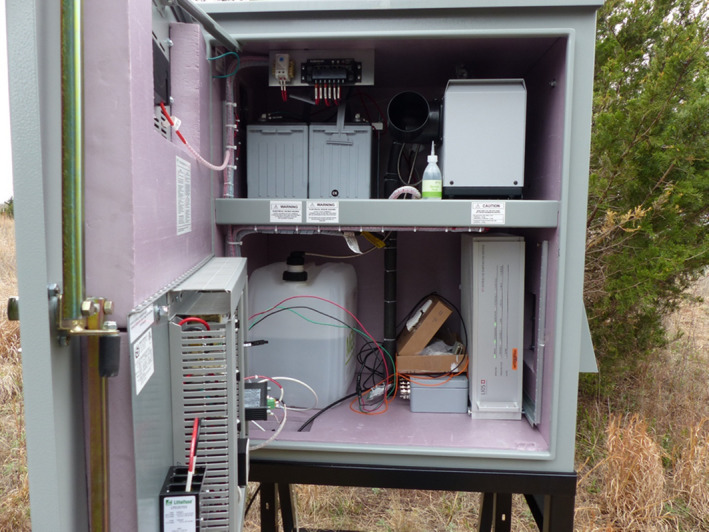
A photograph of the weatherproof housing and fueling system (upper shelf and lower left shelf) that supported our distributed temperature sensing system's controller (lower right shelf) approximately 15 km west of Stillwater, Oklahoma, USA

To quantify changes in the thermal landscape across space and time at fine resolutions, we utilized a Raman‐based fiber optic distributed temperature sensing system. These systems have been shown to continuously measure temperature at resolutions up to 0.01℃ for spatial and temporal resolutions of every meter and under 1 min at distances up to 10,000 m (Selker et al., 2006). For a detailed description on the physics associated with this system, we refer the reader to Appendix 1. We deployed a PRE.VENT 20 distributed temperature sensing system (Linear Optical Sensors, Portland, Oregon, USA) in a duplexed single‐ended configuration (Figure A2). This configuration uses two colocated fibers within a single housing that are fused together at the end of the cable (furthest from the distributed temperature sensing system), which allowed us to obtain two measures of temperature at every point along the cable. Because changes in external temperatures enforced on the distributed temperature sensing system's controller can influence the precision of thermal signatures (Hausner et al., 2011), we housed our system within a weatherproof enclosure affixed with an internal thermostat set to 25℃.

A metal free duplex fiber optic cable with a black protective coating with an outer diameter of 7.0 mm was deployed as a linear transected within the high, medium, and low *J. virginiana* plots. The initial length of the cable was 1 km, however because of external calibration requirements and splicing that was required for cable maintenance, we focused our analysis on a 625 m section of cable that intersected all *J. virginiana* plots (Figure A1). Additionally, lengths within the high (*n* = 259 m), medium (*n* = 223 m), and low (*n* = 100 m) *J. virginiana* density pastures varied based on cable placement. As there is a balance between temperature accuracy and the spatio‐temporal resolutions used for these systems (Hausner et al., 2011), we selected a 1 m and 1 min spatial and temporal resolution for our data collection, respectively. Because our fiber optic cable was contained within a black protective coating, our thermal measurements were synonymous with black body temperatures, which incorporates the effects of temperature, solar radiation, and wind into a thermal index (Porter & Gates, 1969; i.e., operative temperature). By incorporating the effects of solar radiation and wind into a thermal index, we sought to obtain closer approximations of microclimatic conditions that a terrestrial organism might experience at a given ambient temperature across the landscape (Helmuth et al., 2010).

### 2.3 Distributed temperature sensing calibration

Calibration of temperature data through the use of external independent temperature monitors is necessary to obtain accurate temperatures through distributed temperature sensing systems (Hausner et al., 2011). This is because the accuracy of data can vary due to attenuation loss with distance, Raman scattering, “step losses” associated with fiber stress and splices, and instrument sensitivity (Hausner et al., 2011; Tyler et al., 2009). To accomplish this, we assembled two calibration baths (within coolers) at each end of the cable (Figure A1), in which one bath was kept cold by continually adding ice and one bath was kept at the ambient air temperature. The different temperatures in calibration baths were to ensure that we captured the entire potential range of ambient temperatures that our system would be exposed to (Hausner et al., 2011). We submerged approximately 20 m of cable into each calibration bath. Within each bath, we installed an air bubbler to circulate water to help avoid thermal stratification (Hausner et al., 2011). For our external temperature sensor, we deployed a RBRsolo sensor (NIST certified to 0.002℃ accuracy, RBR Ltd, Ottawa, Canada) into each calibration bath to gain highly accurate independent temperature readings for calibration purposes (Cheng et al., 2017). For the theory related to single‐ended calibration of these systems, we refer the reader to (Hausner et al., 2011).

We used dynamic calibration for all temperature measurements during our study by calibrating each temperature trace (i.e., calibrating data every minute for every meter). To assess the accuracy of our calibration procedure, we estimated a root mean square error and a duplexing error (Hausner et al., 2011). The root mean square error assesses the accuracy of the calibration procedure by comparing calibrated temperature to independent calibration bath temperatures (Hausner et al., 2011). The duplexing error assess the error associated with duplexed cables, as fiber optics within the cable housing can move during deployment or after splicing occurs (Hausner et al., 2011). The root mean square error of our data was 0.28℃ while our duplex error was 0.42℃.

### 2.4 Microsite field measurements

We obtained microsite vegetation structural and categorical cover data 1 week following the solar eclipse on a day with similar meteorological conditions. Cover (i.e., vegetation type [*J. virginiana*, deciduous riparian forest, mixed grass, shrub, and bare ground]) categories along the fiber optic cable were recorded to the nearest 10 cm using the line interception method (Canfield, 1941). Vegetation height (cm) was quantified every five meters using a telescoping fiberglass measuring rod. Similarly, we measured photosynthetic active radiation (PAR) every five meters using an AccuPAR LP‐80 ceptometer (Decagon Devices, Pullman, WA). We took 10 PAR measurements directly underneath the vegetation canopy and aligned with the fiber optic cable. We also took 10 PAR measurements in the nearest area from our fiber optic cable with no obscurity from vegetation. For each location, the 10 PAR measurements were averaged for analyses. All PAR readings were conducted during 0% cloud cover and from 11:45–13:45 CDT (local time = UTC – 6 hr). Finally, we obtained downwelling global solar radiation (W/m^2^) measurements occurring during the eclipse from a LI200S pyranometer attached to a permanent weather station (associated with a statewide weather station system [Mesonet]) located approximately 844 m from the DTS station (Brock et al., 1995; McPherson et al., 2007). Solar radiation measurements were recorded every 5 min. Though we were only able to obtain solar radiation from a single point in space in this approach, we used these values to represent the relative overall amount of solar radiation occurring at our study site during the eclipse.

### 2.5 Data analysis

To visualize the thermal signature of our data through space and time, we created a heat map in Program R (version 4.0.1). We estimated the relationships between vegetation cover type (*J. virginiana*, deciduous riparian forest, mixed grass, shrub, and bare ground), vegetation height, and available PAR underneath the vegetation canopy on thermal variance (*n* = 125; measured every 5 m along 625 m cable length) during the eclipse by using generalized least squares models accounting for spatial autocorrelation. First, model performance of additive and interactive models using all three explanatory variables was assessed with five different forms of spatial autocorrelation structure (Gaussian, exponential, linear, ratio, and spherical). These models were compared to a similar model with no autocorrelation structure using Akaike information criterion corrected for small sample sizes (AIC_c_ [Akaike, 1974]) with the package “nlme” (Pinheiro et al., 2017) in Program R (version 4.0.1). We considered models with a ΔAIC_c_ < 2 as plausible models (Burnham & Anderson, 2002). We then incorporated the best performing spatial autocorrelation structure for each model and compared all additive and interactive models adjusted for spatial autocorrelation using AIC_c_ values and estimated psuedo‐*R* ^2^ values to assess the model's predictive capabilities. To quantify structural heterogeneity across high, medium, and low *J. virginiana* density pastures, we estimated means and standard deviations of vegetation height along our cable for each vegetation type (Londe et al., 2020) and compared vegetation height values using a one‐way analysis of variance (ANOVA). If ANOVA results indicated significant differences, we used a post hoc Tukey multiple comparison test (Zar, 2009) to determine differences in height values between pastures at the *p* < .05 level.

To estimate semivariograms, we used the package “gstat” (Pebesma, 2012) in Program R (version 4.0.1) to fit models to our thermal data across the entire length of cable and across the high, medium, and low *J. virginiana* density pastures for every minute, starting 10 min before the eclipse and ending 10 min after. Semivariograms can be fit with different theoretical models (Pebesma, 2012), so we used the “gstat” package to test the fit of spherical, exponential, Matérn's, and the M. Stein's parameterization models (Pebesma, 2012) for every minute of our thermal data during this temporal period. The best fitting model was chosen for each minute of data by comparing the sum of squares error (Pebesma, 2012). To assess spatial autocorrelation across semivariograms, we used the estimated range values for each minute (Rossi et al., 1992). Likewise, to estimate the magnitude of spatial heterogeneity in the thermal data through time, we calculated the partial sill of each semivariograms (Rossi et al., 1992). Finally, we used generalize least squares models to determine the influence of changing solar radiation levels and ambient temperatures on thermal heterogeneity (i.e., partial sills; *n* = 38; estimated every 5 min during the length of the eclipse event). Relationships were estimated for the entire cable length (*n* = 625 m) and for the high (*n* = 259 m), medium (*n* = 223 m), and low (*n* = 100 m) *J. virginiana* density pastures. As the relationship between changes in partial sill values to changes in solar radiation and temperature were a time series associated with the eclipse event, we used the auto.arima function in the package “forecast” (Hyndman et al., 2019) to build models with different time series modeling approaches. These included models with autoregressive components, moving average components, and integration/differencing components. We used default auto.arima settings and the most parsimonious model based on AIC_c_ carried forward in subsequent analyses. Similar to our microsite analyses, we estimated model performance within an information‐theoretic approach using AIC_c_ and considered models with a ΔAIC_c_ < 2 plausible (Burnham & Anderson, 2002) while assessing the ability of our most parsimonious to explain variability in our data using an *r* ^2^ Pearson correlation statistic.

## 3 RESULTS

### 3.1 Change in thermal conditions during the eclipse

**FIGURE 1:**
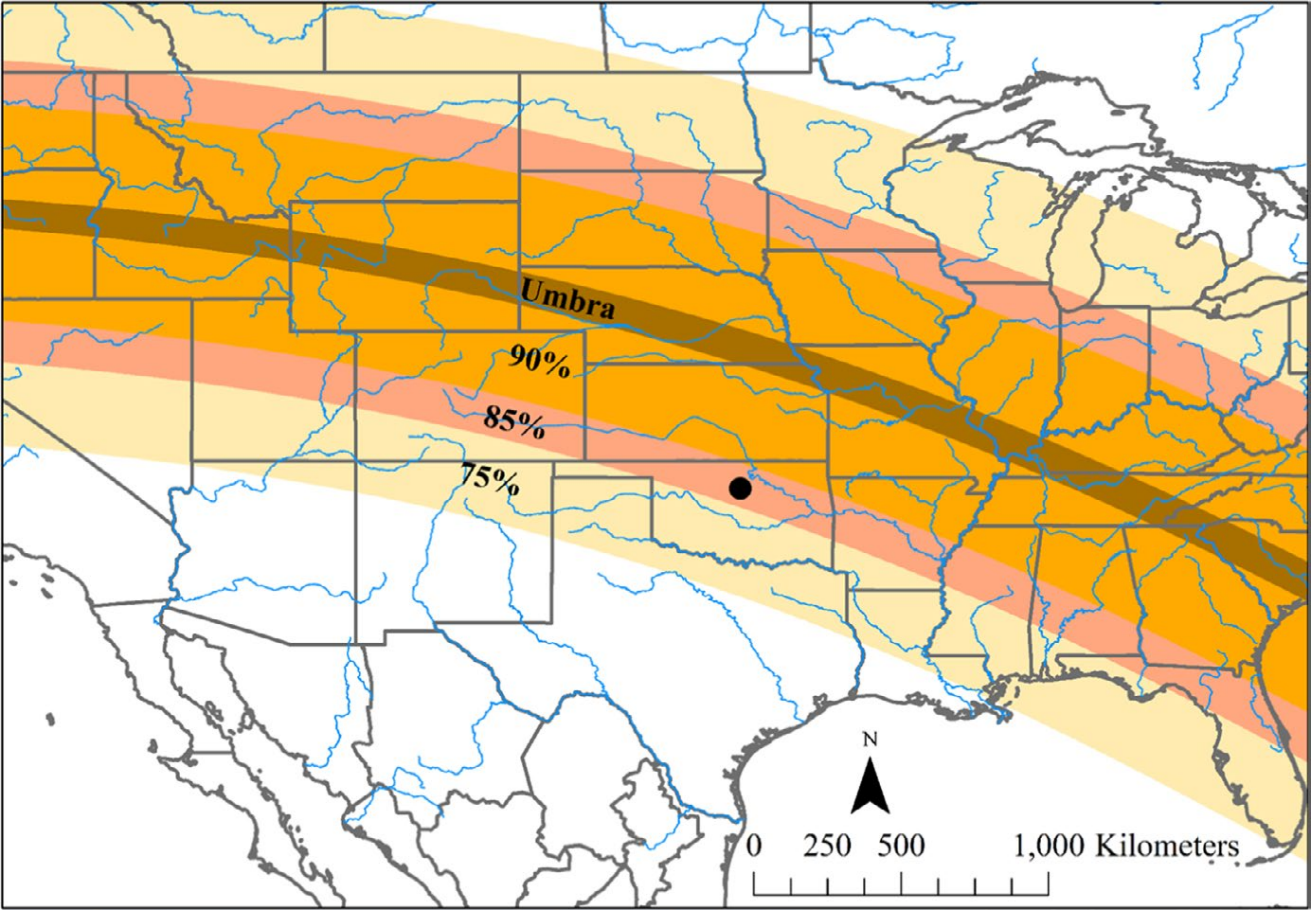
A partial map of the United States showing the zones of umbra (total solar eclipse; brown) and partial solar eclipse (90% [orange], 85% [pink], and 75% [beige]). The black circle indicates the location of the distributed temperature sensing system deployed during the solar eclipse event. Major rivers of North America (blue line) are included for geographic reference. Solar eclipse shape files source: NASA's Scientific Visualization Studio http://svs.gsfc.nasa.gov/4518

**FIGURE A3:**
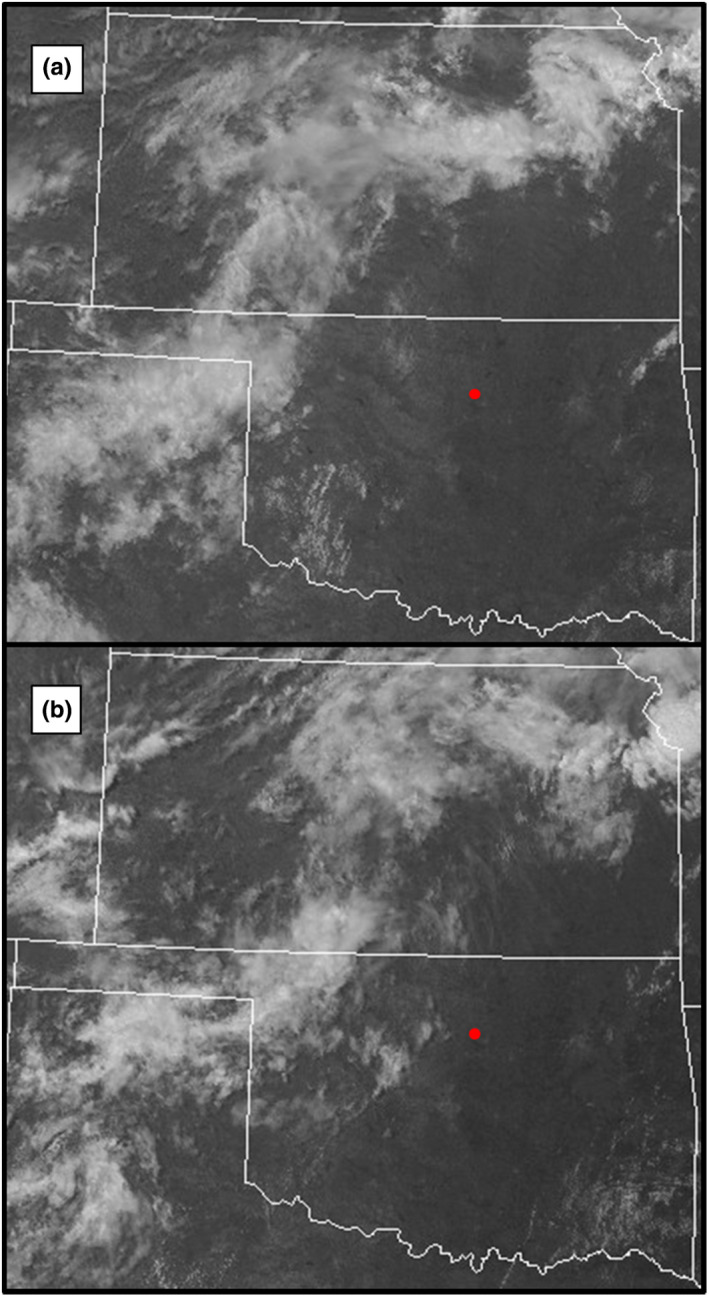
Visible geostationary satellite images taken before (11:45 CDT; A) and after (14:15 CDT; B) the peak of the August 21st, 2017, eclipse. A red circle indicates the location of the study site approximately 15 km west of Stillwater, Oklahoma, USA. Satellite images were originally downloaded from the National Center for Atmospheric Research MMM image archive at http://www2.mmm.ucar.edu/imagearchive/. Original satellite images were obtained from the National Oceanic and Atmospheric Administration's Aviation Weather Center (https://www.aviationweather.gov/) using Geostationary Operational Environmental Satellite‐R Series satellites

The total solar eclipse began at 11:37 Central Standard Time (CDT) was at its maximum totality at 13:06, and ended at 14:35 at our study site. Due to the latitude and longitude of our study site, the maximum coverage of the sun was estimated as 87.32% and had a magnitude of 0.8937 (Figure 1). Directly before, during, and directly after the eclipse, the sky above our study site was cloud‐free (Turner et al., 2018; Figure A3).

**FIGURE 2:**
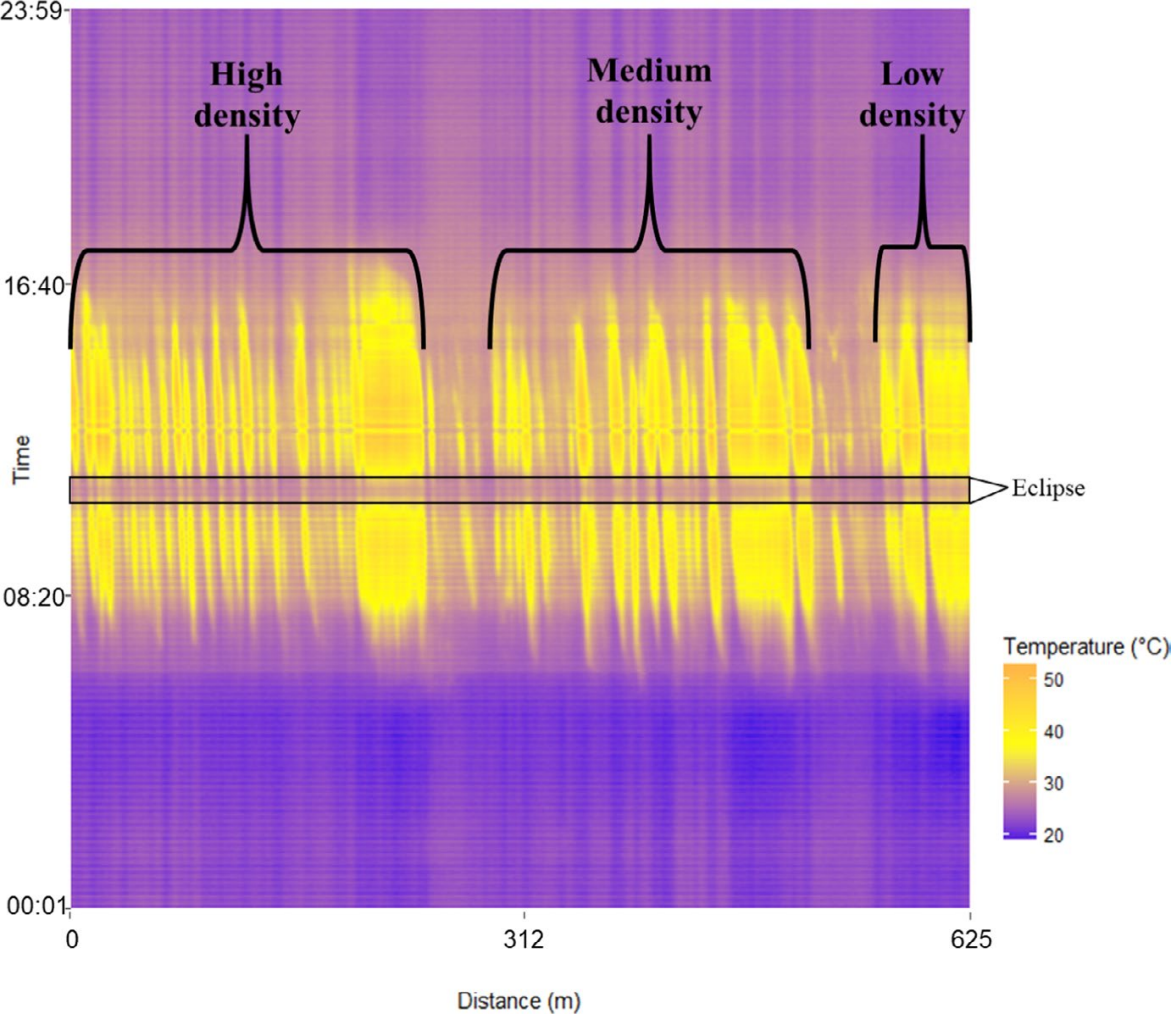
A heat map indicating the thermal conditions recorded by a distributed temperature sensing system along a fiber optic cable (625 m in length) during the August 21st, 2017, solar eclipse in Stillwater, Oklahoma, USA. The spatial and temporal resolution of the thermal data were 1 m and 1 min, respectively. A black box outlines the homogenization of the thermal landscape during the midpoint of the solar eclipse event. Pastures with varying levels of *Juniperus virginiana* density (high, medium, and low) are indicated in the heat map. Densities are: high (~60% *J. virginiana* cover/cable meter), medium (~46% *J. virginiana* cover/cable meter), and low (~16% *J. virginiana* cover/cable)

The minimum ambient temperature during the eclipse (30.56℃) exhibited a lag effect in response to changes in solar radiation levels, a phenomenon typical of eclipse events (Good, 2016; Turner et al., 2018) and particularly of this eclipse event (Buban et al., 2019). For example, the minimum ambient temperature occurred from 13:15 to 13:30 CDT (Figure 2), yet the minimum value of downwelling global solar radiation (hereafter: solar radiation; 90 W/m^2^) occurred at approximately 13:10 CDT. The maximum ambient temperature observed during the eclipse (33.33℃) occurred at 12:00 CDT and 14:30–14:35 CDT. Conversely, the maximum black body temperature recorded along the fiber optic cable (52.59℃) during the eclipse coincided with the maximum ambient temperature at 14:35 CDT while the minimum temperature (25.08℃) coincided with the minimum value of solar radiation observed at 13:10 CDT. Both the maximum and minimum temperatures recorded along the fiber optic cable occurred in the mixed grass vegetation type.

Overall, the greatest temperature range (i.e., maximum temperature – minimum temperature [*T^r^*]) in black body temperatures during the eclipse occurred within mixed grass vegetation, while *J. virginiana* canopies exhibited the least amount of change suggesting a higher thermal buffering effect (Figure 4). Variance in temperature ranges (Var[*T^r^*]) during the eclipse was lowest for the mixed grass cover type (Var[*T^r^*] = 11.9), compared to *J. virginiana* (Var[*T^r^*] = 15.6), deciduous riparian (Var[*T^r^*] = 17.2), and bare ground (Var[*T^r^*] = 18.5) cover types along the cable.

**FIGURE 3:**
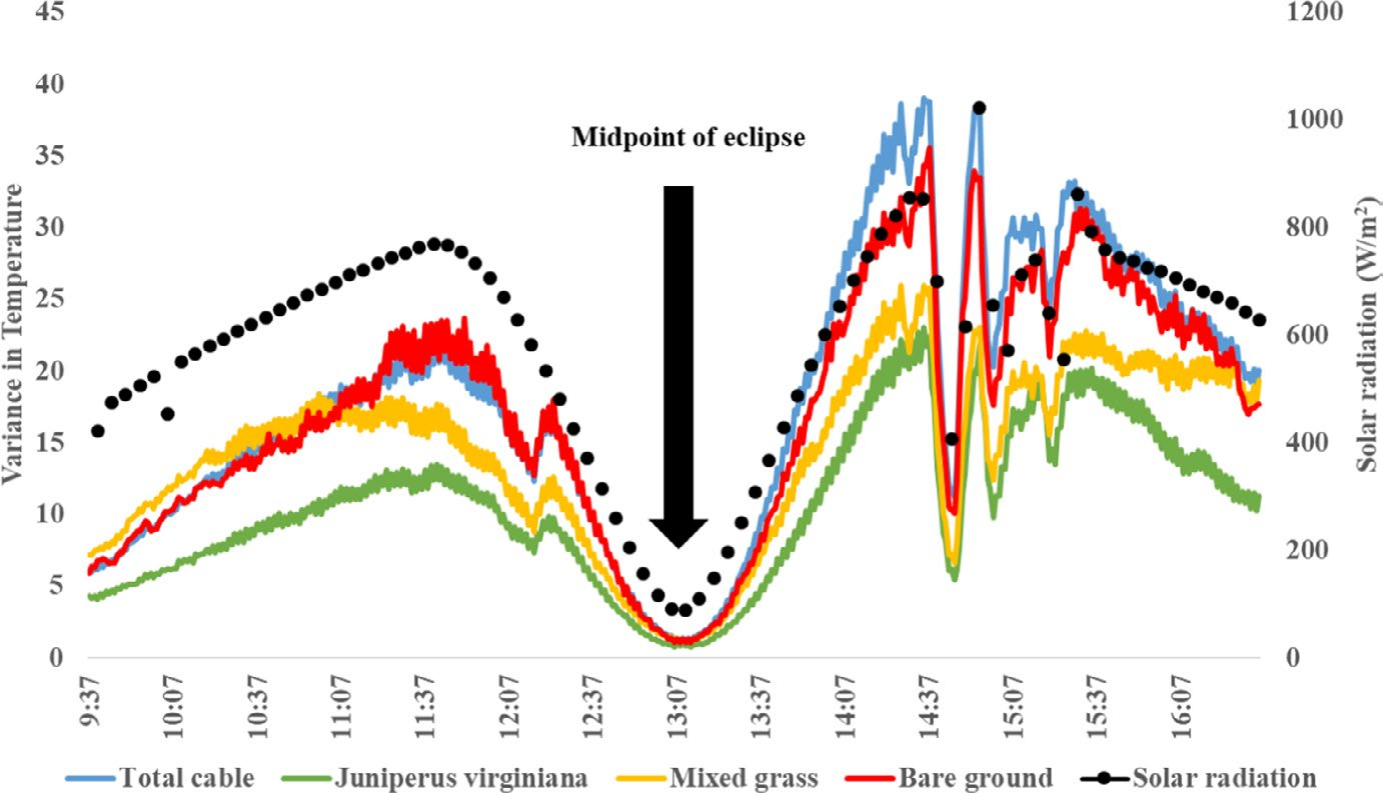
Variance in temperature through time as measured by a distributed temperature sensing system along a 625 m fiber optic cable during a solar eclipse in Stillwater, Oklahoma, USA. Overall thermal variance (blue) and thermal variance of different cover types (bare ground [red], mixed grass [yellow], and *Juniperus virginiana* [green]) were calculated every minute. Solar radiation (black circle) was estimated every 5 min by a weather station approximately 844 m from the distributed temperature sensing system

Variance in black body temperatures across the fiber optic cable was correlated with the amount of incoming solar radiation during the eclipse (first differences correlation |*r*| = 0.66). Thermal variance was greatest within bare ground patches and lowest within *J. virginiana* canopies (Figure 3), though only mixed grass vegetation had significantly higher thermal variance compared to *J. virginiana* and deciduous riparian vegetation types (*p* <.05). However, variance across all vegetation types nearly homogenized during the midpoint of the solar eclipse (temperature variance range: 0.8–1.1). Patterns in thermal variance during the eclipse were not only related to vegetation type, but also were significantly related to vegetation height (cm; *p* =.006) and the relative amount of photosynthetic active radiation underneath the vegetation canopy (µmol; *p* =.002; Tables A1 and A2). Vegetation height was greatest in the *J. virginiana* vegetation type ($\bar{x}$ = 283.03 cm, *SE* = 27.85), followed by deciduous riparian ($\bar{x}$ = 222.40 cm, *SE* = 107.53), mixed grass ($\bar{x}$ = 91.67 cm, *SE* = 19.54), and shrub ($\bar{x}$ = 73.46 cm, *SE* = 48.46). We note that no bare ground patches were included in the height analysis, as they did not fall along the 5 m interval of our sampling design. Both high ($\bar{x}$ = 228.22 cm, *SD* = 207.19) and medium ($\bar{x}$ = 186.86 cm, *SD* = 207.19) *J. virginiana* density pastures had high vegetation height heterogeneity compared to the low *J. virginiana* density pasture ($\bar{x}$ = 56.97 cm, *SD* = 65.42). One‐way ANOVA results indicated differences in vegetation heights within high, medium, and low *J. virginiana* density pastures (*p* = .025). Specifically, there was a significant difference in vegetation heights between high and low *J. virginiana* density pastures (*p* = .019), though no significant differences were found between high and medium (*p* = .55) and medium and low (*p* = .08) *J. virginiana* density pastures.

### 3.2 Thermal heterogeneity ranges during the eclipse

Semivariograms indicated that, when compared to the midpoint of the eclipse, the magnitude of thermal heterogeneity across the entire cable (i.e., partial sill values [Rossi et al., 1992]) was 15.4 and 30.4 times greater 10 min before and after the eclipse, respectively (Extended Figure 4). Changes across time in the magnitude of thermal heterogeneity at our study site was directly related to the amount of solar radiation being blocked throughout the eclipse (*p* = <0.001, *r* ^2^ = 0.98; Figure 5; Table A3). Despite this trend, changes in the spatial autocorrelation of thermal conditions (semivariogram range values) during the eclipse were less marked, with the range of semivariograms having lag distances between 72.7 and 90.5 m.

**FIGURE 4:**
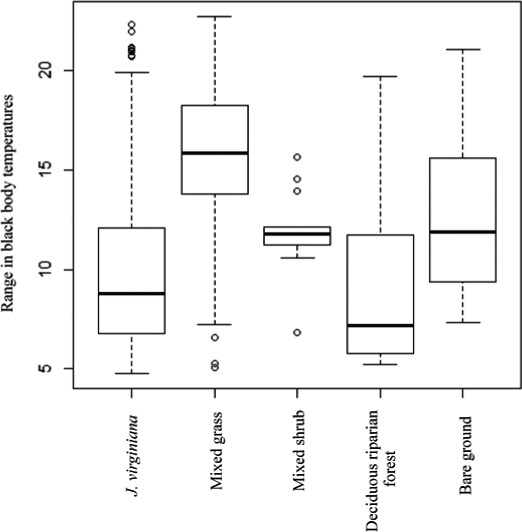
Box plots indicating the overall change in thermal conditions by cover type during the August 21st, 2017, solar eclipse in Stillwater, Oklahoma, USA. Range represents the absolute value of the difference between the maximum and minimum temperatures recorded during the solar eclipse by cover type. Temperature were recorded for every meter and every minute along a 625 m fiber optic cable through the use of a distributed temperature sensing system

**FIGURE 5:**
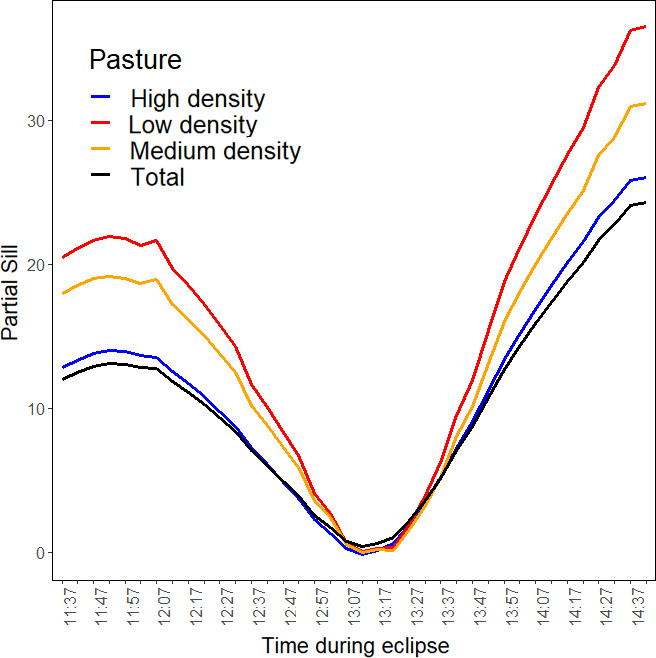
Time series models predicting changes in partial sill values of semivariograms representing spatial heterogeneity of thermal conditions as a function of changes in solar radiation and ambient temperature during the August 21st, 2017, solar eclipse in Stillwater, Oklahoma, USA. Semivariograms were estimated for thermal conditions along a 625 m fiber by a distributed temperature sensing system for the entire cable length (“total”; black line), and within pastures with high (blue line), medium (orange line), and low (red line) *Juniperus virginiana* densities. Temperatures were recorded with 1 m and 1 min spatial and temporal resolutions, respectively. Solar radiation was estimated every 5 min by a weather station approximately 844 m from the distributed temperature sensing system

**FIGURE 6:**
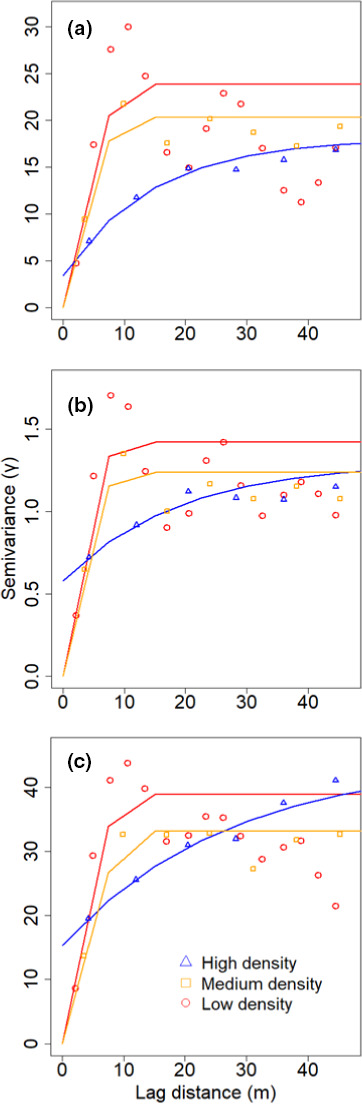
Estimated semivariograms for temperatures measured within pastures with high (blue), medium (orange), and low (red) density *Juniperus virginiana* encroachment along a 625 m fiber optic cable by a distributed temperature sensing system. Semivariograms were estimated 10 min before (11:27 Central Standard Time; a), during the midpoint period (13:05 Central Standard Time; b), and 10 min after (14:45 Central Standard Time; c) a solar eclipse in Stillwater, Oklahoma, USA on August 21st, 2017

Patterns in vegetation cover along the fiber optic cable influenced the magnitude of thermal heterogeneity occurring across our study site (Figure 6). Based on partial sill values across changes in solar radiation, the pasture with low‐density *J. virginiana* consistently had greater thermal heterogeneity compared to medium and high‐density *J. virginiana* pastures (Figure 5). The absolute change in partial sill values was greatest for low‐density *J. virginiana* pasture (Δ partial sill = 37.47), intermittent for medium‐density pasture (Δ partial sill = 31.95), and lowest for the high‐density pasture (Δ partial sill = 13.37). Similar to patterns across the entire cable, the amount of solar radiation influenced changes in thermal heterogeneity for each pasture (*p* < .01; Table A3). The *β* estimates from these models suggested the greatest change in thermal heterogeneity in relationship to a change in solar radiation occurred within the low‐density pasture (*β* = 0.04, *p* < .01), while the medium density (*β* = 0.03, *p* < .01) and high‐density (*β* = 0.03, *p* < .01) pastures had smaller effect sizes. Although ambient temperature was included in plausible models explaining changes in thermal heterogeneity (Table A3), it was not significant for the medium (*p* = .38) or low (*p* = .27) *J. virginiana* pastures. Similarly, the univariate ambient temperature model carried no weight for any pasture nor for the entire cable (Table A3).

## 4 DISCUSSION

Our research utilized an astronomical event with a novel method to help understand how structural heterogeneity inherent in natural landscapes can influence the thermal landscape. Specifically, we were able to utilize this event as an alteration of ultimate forces (incoming solar radiation) to mimic a rapid sunrise/sunset event to explicitly quantify the changes in temperatures and spatial variability of diurnal thermal conditions (i.e., thermal heterogeneity) associated with woody encroachment in a standardized approach. Ultimately, this experiment illustrated that the greatest simulated diurnal temperature range occurred within the mixed grass vegetation type, while the pasture with the lowest amount of woody encroachment had the greatest amount of thermal heterogeneity and exhibited the largest overall diurnal change in thermal heterogeneity. Though the pasture with the highest amount of woody encroachment had the smallest simulated diurnal range of thermal heterogeneity, spatial variability of thermal conditions was also lowest in this pasture throughout all levels of incoming solar radiation. Thus, our results suggest that highly encroached grasslands may experience lower diurnal variability of temperatures at the cost of a decrease in the overall thermal heterogeneity of that landscape.

However, we note that this relationship is scale specific and related to thermal conditions measured underneath plant canopies (Geiger, 1965). For instance, patterns in our study relate to near‐surface temperatures quantified every meter and every minute along a linear transect within the buffering layer of vegetation cover (proximate influences). At a local microsite level, woody plants can create thermal buffers within their canopy by influencing long‐wave radiation interactions between the ground's surface and vegetation structure (D’Odorico et al., 2013). However, at a much larger extent, ultimate forces such as changes in land surface albedo could have opposite effects on thermal conditions within an encroached grassland. For instance, encroachment of *J. virginiana* can lower the overall albedo of an invaded grassland, thus increasing the net radiation, resulting in higher sensible and latent heat flux, and ultimately increasing temperatures in woody encroached grasslands (Ge & Zou, 2013). These disparate relationships illustrate the importance in understanding the complex relationships between ultimate and proximate forces that drive thermal conditions in dynamic landscapes.

Structural heterogeneity (i.e., the existence of variable vegetation types and vegetation structure) in landscapes is a key mechanism for facilitating ecosystem functions and providing organisms with multiple options for abiotic and biotic conditions and resources through space and time (Turner et al., 2001). Thermal landscapes are inherently determined by many factors that structure heterogeneity across spatial and temporal scales and are thus highly dynamic in nature (Aalto et al., 2013; Carroll et al., 2015; Saunders et al., 1998; Sears & Angilletta, 2015; Sears et al., 2016). As landscapes continue to be altered by humans globally, the related structural changes will effectively alter the spatio‐temporal trends of thermal landscapes concurrently (Tuff et al., 2016). For instance, within our study system, changes in historical fire regimes have facilitated ecological processes such as woody encroachment, a phenomenon which is occurring at a global scale across most grassland systems (Bond, 2008). The spread of *J. virginiana* into the North American Great Plains has already greatly altered biotic communities and abiotic conditions, and our data suggest that this ecological process is simultaneously altering thermal spatio‐temporal patterns within grasslands (Engle et al., 2008). The low‐density *J. virginiana* pasture consistently had greater spatial variance in thermal patterns throughout the eclipse, suggesting it provided greater thermal heterogeneity than the medium‐ and high‐density pastures (Figures 5 and 6). This is consistent with previous literature exploring PAR reaching the ground level within a grassland‐forest continuum (Martens et al., 2000), in which PAR variance in the understory exhibits a curvilinear relationship with the greatest PAR variance occurring between ~30‐ and 45% (Martens et al., 2000). Given that grasslands have been shown to transition to woodlands at low thresholds (~18.5% cover [Loehle et al., 1996]) and considering that our low‐density pasture had ~16% *J. virginiana* cover, our data illustrate the potential magnitude of change that can occur within a thermal landscape once grasslands begin to transition to woodlands.

This alteration in thermal spatio‐temporal patterns associated with woody encroachment may become a conservation conundrum. The ability to buffer thermal extremes is critical in influencing the reproductive success (Hovick et al., 2014; Raynor et al., 2018) and space use (Kauffman et al., 2021; Londe et al., 2021) of many grassland obligate species in the Great Plains. Moreover, many of these species exhibit negative responses to woody encroachment across multiple scales that could lead to local extinctions (Fuhlendorf et al., 2002). However, local extinctions can also been linked to thermal extremes (Sinervo et al., 2010) and near‐surface thermal conditions within woody canopies may provide thermal refuge during critical periods (pulse dynamics) at the cost of long‐term habitat degradation (press dynamics). Given that areas that characteristically have high heat and periodic aridity are predicted to experience some of the greatest increases in thermal conditions under future climatic scenarios (Meehl & Tebaldi, 2004), woody encroached grasslands may become a system of ecological trade‐offs within the auspices of thermal stressors.

We observed an absolute range of 27.08℃ in thermal conditions across our fiber optic cable during the eclipse. Furthermore, we observed a maximum decrease of 22.76℃ within a single meter on the fiber optic cable during this event. This maximum decrease was associated with a mixed grass vegetation type, similar to a 15.5℃ decrease in grassland temperatures reported during the 1999 total solar eclipse throughout the United Kingdom (Hanna, 2000). Moreover, Hanna (2000) reported substantial decreases in ground temperatures within grasslands throughout sites across the United Kingdom during the 1999 eclipse, despite sample sites experiencing varying percentages of totality. It is clear that there is a high degree of variability between temperature decreases reported across eclipse studies (Good, 2016). The large decrease in temperatures reported in our study relates to the measurement of the black body temperature (i.e., operative temperature of an organism). This thermal index allowed us to quantify the spatio‐temporal thermal gradient as it pertains to the interaction between solar radiation and land cover, thus offering an estimation of how thermal landscapes mitigate conditions for organisms.

We expected that there would be differences in the variability of temperatures across vegetation types during the eclipse because different vegetation types can mitigate thermal conditions through disparate structural and biological properties (Londe et al., 2020; Stoutjesdijk & Barkman, 1987). For instance, the volume and percent cover of shrubs can directly influence the diurnal temperature range that an area experiences, in which increased shrub height, canopy width, and shrub cover results in lower ranges and more stable thermal environments (Anthony et al., 2020; Milling et al., 2018). *J. virginiana* canopies consistently had the lowest variability throughout the eclipse, which was driven by vegetation height and the amount of photosynthetic active radiation being blocked by the vegetation's canopy (Tables A1 and A2). Conversely, bare ground and mixed grass cover types exhibited considerably higher variability throughout the eclipse, excluding the midpoint of the eclipse. These results highlight the importance that the “outer active surface” (i.e., the surface in which maximum temperatures occur within a plant canopy [Geiger, 1965]) play in structuring thermal heterogeneity. As plant density can shift the outer active surface from the ground layer to the top surface of the vegetation layer (Geiger, 1965), ground surface temperatures within *J. virginiana* canopies inherently had lower thermal variability.

Utilizing total solar eclipse events to document changes in floral, faunal, and ecosystem patterns have largely been housed within anecdotal accounts (Buckley et al., 2018) though can offer unique opportunities to understand how solar radiation structures ecological phenomena (Gil‐Burmann & Beltrami, 2003; Kullenberg, 1995). Within the context of thermal landscapes, trends observed during the eclipse can highlight the ecological implications of shifting cover types associated with woody encroachment based on the thermal conditions that are being created through the presence of these cover types. For instance, based on the thermal signatures mapped throughout the 24‐hr period, it is evident that areas of mixed grass and bare ground have higher temperatures during the peak of the day, though cool off at a faster rate than underneath *J. virginiana* and riparian mixed‐forest canopies (Figure 2). This same trend was evident during the midpoint of the eclipse as well. Thus, our data support the idea of forest canopies buffering thermal extremes within grassland systems. Such shifts in thermal signatures can have important implications for ecological patterns and processes such as influencing the frequency of preferred microclimates for organisms or altering soil respiration rates (Krause et al., 2013; Sears et al., 2016; Smith & Johnson, 2004). These trends further substantiate the importance that cover types, and the patterns of heterogeneity created by these cover types, have on the thermal patterns exhibited during astronomical events such as a solar eclipse (Good, 2016).

Ultimately, the occurrence of a solar eclipse allowed us to explicitly quantify the changes in thermal heterogeneity through space and time along a continuum by observing differing levels of solar radiation present in the environment that mimicked a rapid sunset/sunrise event. The highly encroached grassland exhibited the lowest diurnal change in thermal heterogeneity at the cost of having the lowest thermal heterogeneity regardless of the amount of solar radiation present in the system. Conversely, the grassland with the least amount of woody encroachment consistently had the highest amount of thermal heterogeneity and experienced the largest range of heterogeneity values. This thermal heterogeneity that is facilitated by inherent patterns in landscapes will continue to be a crucial ecosystem function as species face the potential negative influences of global climate change (IPCC, 2014; Pincebourde et al., 2016; Thomas et al., 2004; Walther et al., 2002). Microclimatic variability may help to stabilize local populations that would otherwise be forced to shift their distributions or risk extinction (Hampe & Petit, 2005; Sears & Angilletta, 2015). By quantifying the spatio‐temporal changes in thermal heterogeneity and the magnitude of differences in heterogeneity that occur within a single landscape through the lens of an astronomical event, the importance of scaling in both space and time are ascertained for thermal landscapes within the scope of future conservation biology.

## CONFLICT OF INTEREST

None declared.

## AUTHOR CONTRIBUTIONS

**Evan P. Tanner:** Conceptualization (equal); data curation (equal); formal analysis (equal); investigation (equal); methodology (equal); project administration (equal); visualization (equal); writing–original draft (equal); writing–review and editing (equal). **Samuel D. Fuhlendorf:** Funding acquisition (equal); investigation (equal); methodology (equal); project administration (equal); writing–original draft (equal); writing–review and editing (equal). **John Polo:** Investigation (equal); methodology (equal); writing–original draft (equal); writing–review and editing (equal). **Jacob M. Peterson:** Formal analysis (equal); writing–original draft (equal); writing–review and editing (equal).

## Figures and Tables

**FIGURE A4:**
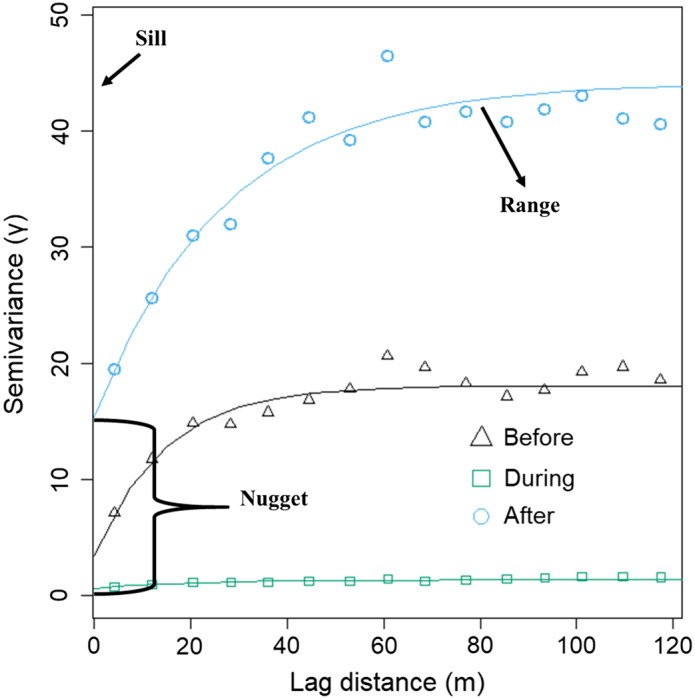
Estimated semivariograms for temperatures measured along a 625 m fiber optic cable by a distributed temperature sensing system. Semivariograms were estimated 10 min before (11:27 Central Standard Time; black), 10 min after (14:45 Central Standard Time; blue), and during the midpoint period (13:05 Central Standard Time; green) of a solar eclipse in Stillwater, Oklahoma, USA on August 21st, 2017. Key elements of a standard semivariogram are indicated for the semivariogram estimated during the period 10 min after the solar eclipse. These include range—the lag distance in which the semivariogram first flattens, illustrating that samples located closer together than this distance are spatially autocorrelated; sill—the value of semivariance in which the model reaches the range; nugget—an estimate of spatial sampling error

## Data Availability

Data generated from this study are deposited online in Dryad and available at https://doi.org/10.5061/dryad.t4b8gtj2d.

## APPENDIX 1

### Distributed Temperature Sensing Description

This system sends laser pulses through the quartz core of a fiber optic cable and quantifies the proportion of energy from the laser pulse that is returned to the system's receiver, which is recorded as optical backscatter (Krause et al., 2013). To quantify thermal conditions, the system compares a dynamic amplitude ratio of Stokes/anti‐Stokes along with a time‐of‐travel metric from the laser pulse's system (Krause et al., 2013). This system incorporates Rayleigh and Raman scattering theories. Rayleigh scattering refers to elastic collisions between molecules and photons within the fiber's quartz core, which allow for the return of the majority of backscatter to return to the receiver at the laser pulse's original frequency. Raman scattering refers to inelastic collisions between photons and electrons, which will return the laser pulse's signal to a frequency that is either lower (Stokes) or higher (anti‐Stokes) than the original pulse frequency (Tyler et al., 2009). Temperature acts as an external force on the quartz core to facilitate a high energy state for electrons, in which higher temperatures results in more electrons in high energy states, thus increasing the amount of scattered anti‐Stokes compared to Stokes (Tyler et al., 2009). By incorporating these theories, the amplitude ratio of Stokes/anti‐Stokes provides a measure of temperature at a location along a fiber based on the two‐way travel time of the laser pulse starting from its initial light injection (Hausner et al., 2011; Tyler et al., 2009).

**TABLE A1:** Model selection results for influences of vegetation type, vegetation height (height; cm), and photosynthetic active radiation (PAR; µmol) on thermal variance during the August 21st, 2017, solar eclipse approximately 15 km west of Stillwater, Oklahoma, USA

Model	*K* ^a^	AIC_c_	ΔAIC_c_	Cumulative weight	Log‐restricted likelihood	Psuedo‐*R* ^2^	Correlation Structure
Vegetation + height + PAR	8	839.82	0	0.36	−411.29	0.50	None
Vegetation + PAR	9	839.83	0.01	0.72	−410.13	0.47	Ratio
Vegetation	8	841.30	1.48	0.88	−412.03	0.37	Exponential
Vegetation + height	9	841.93	2.11	1.00	−411.18	0.46	Linear
Vegetation × height	11	863.08	23.26	1.00	−419.37	0.50	None
Height + PAR	4	868.35	28.53	1.00	−430.01	0.40	None
Vegetation × PAR	11	873.91	34.09	1.00	−424.79	0.47	None
PAR	3	874.90	35.08	1.00	−434.35	0.30	None
Height	3	881.03	41.21	1.00	−437.41	0.26	None
Height × PAR	5	885.36	45.54	1.00	−437.43	0.43	None
Null	4	889.85	50.03	1.00	−440.76	‐	Spherical

^a^ Number of parameters in model.

**TABLE A2:** Parameter estimates of top model explaining influences of vegetation characteristics on thermal variance

Parameter	Parameter estimate	*SE*	*p* ^a^
Intercept	8.89	1.42	<0.001
Mixed grass^b^	7.20	1.52	<0.001
Shrub^b^	−0.63	4.75	0.89
Deciduous riparian^b^	−0.54	2.68	0.84
Bare ground^b^	3.02	2.63	0.25
PAR^c^	0.01	0.002	0.002
Height^d^	−0.01	0.003	0.006

^a^ *α* = 0.05

^b^ Parameter estimates are in reference to the *Juniperus virginiana* vegetation type.

^c^ Photosynthetic active radiation (µmol).

^d^ Vegetation height (cm).

**TABLE A3:** Model selection results for the influence of solar radiation (W/m^2^) and ambient temperature (°C) on thermal heterogeneity (partial sill) during the August 21st, 2017, solar eclipse approximately 15 km west of Stillwater, Oklahoma, USA

Model	*K* ^a^	AICc	AICc	Cumulative weight	Log‐restricted likelihood	*r* ^2^
*Total landscape*						
Solar radiation + temperature	9	120.76	0	0.68	−61.38	0.98
Solar radiation	8	122.32	1.56	1	−62.16	0.97
Solar radiation × emperature	10	131.19	10.43	1	−93.19	0.98
Temperature	8	155.85	35.09	1	−66.59	0.80
Null	7	179.62	58.86	1	−90.81	–
*High‐density Juniperus virginiana*						
Solar radiation + temperature	9	128.10	0	0.67	−65.05	0.97
Solar radiation	8	129.54	1.44	1	−65.77	0.97
Solar radiation × temperature	10	138.48	10.38	1	−70.24	0.97
Temperature	8	155.82	27.72	1	−78.91	0.27
Null	7	157.46	29.36	1	−79.73	–
*Medium‐density J. virginiana*						
Solar radiation + temperature	7	134.50	0	0.89	−68.25	0.97
Solar radiation	6	138.84	4.34	1	−70.42	0.97
Solar radiation × temperature	8	144.89	10.39	1	−73.44	0.97
Temperature	6	173.05	38.55	1	−87.53	0.82
Null	5	799.51	65.01	1	−100.75	–
*Low‐density J. virginiana*						
Solar radiation + temperature	7	145.52	0	0.92	−73.76	0.97
Solar radiation	6	150.59	5.07	0.99	−76.30	0.97
Solar radiation × temperature	8	155.82	10.29	1	−78.91	0.97
Temperature	6	185.5	39.98	1	−93.75	0.81
Null	5	211.51	65.99	1	−106.76	–

^a^ Number of parameters in model.
